# Exploring Cultural Evolution Through Modular Dynamics in Temporal Hashtag Networks

**DOI:** 10.3390/e28040398

**Published:** 2026-04-01

**Authors:** Yasuhiro Hashimoto, Hiroki Sato, Takashi Ikegami

**Affiliations:** 1 School of Computer Science and Engineering, The University of Aizu, Aizuwakamatsu 965-8580, Fukushima, Japan; 2 Graduate School of Arts and Sciences, The University of Tokyo, Meguro, Tokyo 153-8902, Japan; hsato@sacral.c.u-tokyo.ac.jp (H.S.); ikeg@sacral.c.u-tokyo.ac.jp (T.I.)

**Keywords:** cultural evolution, social media, network clustering, core-periphery structure, temporal networks, ensemble methods

## Abstract

Social media platforms offer unprecedented opportunities to study cultural evolution by analyzing digital traces. This study presents a methodological framework for analyzing the temporal dynamics of cultural modules in hashtag co-occurrence networks. We address the inherent challenges of analyzing dense, skewed, and highly variable cultural networks by introducing a perturbation ensemble clustering approach that distinguishes stable from unstable structural elements. By applying the Leiden algorithm to a perturbed ensemble of hashtag networks, we identify robust core modules and their stable periphery, and distinguish them from floating elements with unstable associations. Analysis of four years of data from a major photo-sharing platform reveals complex patterns in the evolution of cultural modules, including both stable associations and dynamic reorganizations. Our findings demonstrate how ensemble clustering techniques can effectively capture the interplay between stability and change in evolving cultural systems.

## 1. Introduction

Social media has served as a valuable research platform for studying cultural evolution, as it captures the spread and mutation of cultural elements in real time [1,2]. Although early research on digital cultural evolution focused on tracking individual aspects, such as memes [3], subsequent theoretical work has begun to illuminate how cultural and technological elements evolve and recombine over time [4]. In the context of web services and social tagging, prior work has explicitly framed tag combinations and their continual recombination as instances of open-ended evolution, quantifying the emergence of novelties in tag ecosystems [5,6] and exploring evolutionary search processes in hashtag spaces on social network services [7]. Recent large-scale analyses of visual memes have similarly quantified the diversification and evolutionary trajectories of online cultural elements [8]. Together, these studies suggest that digital platforms provide an opportunity not only to observe isolated cultural instances but also to identify broader patterns of cultural transmission and transformation through systematic associations among cultural elements.

Among the various forms of online cultural markers, hashtags and their co-occurrence patterns offer particularly rich data for investigating these dynamics. In this study, we analyze yearly hashtag co-occurrence networks to examine how cultural associations emerge, stabilize, and reorganize over time. Previous studies have shown that different types of content can exhibit distinct patterns of social contagion [9] and that diffusion across communities is strongly related to virality [10]. Competition for limited attention may also shape adoption patterns [11]. In addition, research on social tagging has consistently revealed stable regularities in how users organize and share information [12]. Network approaches to hashtag co-occurrence therefore provide a powerful means of studying cultural organization in digital spaces.

Recent work has increasingly focused on identifying modular structures within cultural networks in order to map the higher-level organization of cultural discourse. Studies have successfully delineated distinct cultural domains in online communication networks [13] and traced how cultural communities form, shift, and evolve within social media discussions [14]. Analyses of core-periphery structures [15,16,17] have further highlighted how cultural systems maintain overall stability even as their constituent elements change. At the same time, mesoscale roles such as cores and connectors in attention networks may be shaped not only by endogenous interaction dynamics but also by broader exogenous conditions, including economic output and geographic proximity [18]. However, these analyses face persistent methodological challenges. Cultural networks are inherently unstable: they are characterized by weak and transient associations, joint usage patterns, and rapid shifts in collective attention [19], which makes it difficult to identify stable cultural modules over time reliably, especially given known limitations of modularity-based community detection such as the resolution limit [20].

Several approaches exist for handling unstable or noisy network structures, including consensus clustering [21] and perturbation ensemble methods [22]. In the broader context of dynamic community detection, deep learning approaches combined with evolutionary clustering have been proposed to capture smoothly evolving community structure [23]. More recently, community detection frameworks enhanced by machine learning have incorporated micro- and mesoscopic statistical physics features to improve partition accuracy and interpretability [24]. However, these approaches generally aim to recover a single well-defined partition, often under assumptions of smoothly evolving structure or in settings where task-specific supervision or externally validated labels are available. By contrast, in cultural co-usage networks, no definitive ground-truth community assignment exists, and weak or context-dependent associations are abundant. We therefore treat assignment uncertainty itself as an object of analysis, adopt a coarse yearly resolution with four temporal snapshots, and use ensemble clustering to assess the robustness of clustering patterns under perturbed edge weights. A key methodological gap, therefore, is how to identify which parts of the yearly hashtag modules are robust to small perturbations and which node assignments remain intrinsically uncertain in dense cultural networks.

To address this gap, we introduce a clustering method based on a perturbation ensemble that distinguishes stable from unstable structural components in temporal hashtag networks. Our aim is not to propose a universally superior community detection algorithm or to maximize a single score for partition quality. Rather, we use repeated perturbations of the underlying weighted networks to quantify the reproducibility of module structure in noisy cultural networks. We apply the Leiden algorithm [25] to each perturbed realization and summarize the resulting ensemble of partitions as module assignment vectors for individual nodes and pairwise frequencies of being assigned to the same module. Accordingly, we use Leiden here as a representative and well-established base partitioner for large weighted graphs, and focus on robustness across perturbed realizations within this partitioning framework rather than on benchmarking alternative community detection algorithms.

Our approach is informed by the literature on temporal networks and dynamic community discovery [26,27]. Instead of imposing an explicit temporal coupling over many time steps (e.g., multislice modularity), we use yearly snapshots and quantify robustness using a perturbation ensemble [21,22,28]. This method identifies stable structures by first extracting core modules—subgraphs that reliably cluster together across perturbed realizations—and then delineating the periphery that remains consistently attached to these cores. Hashtags that do not robustly belong to any core-periphery structure are separated as floating hashtags, whose module affiliations are weaker and more fluid. Accordingly, this study asks three questions: Can a perturbation ensemble approach distinguish robust from unstable modular structures in yearly hashtag networks? How can stable structural roles such as core, periphery, and floating elements be operationalized from ensemble variability? And how do these structural roles evolve across yearly network snapshots?

Our primary methodological contribution is a framework for extracting a robust module structure under weight uncertainty in temporal hashtag networks. Our primary theoretical contribution is to characterize observed cultural organization on online platforms as the coexistence of stable cores and fluid boundaries rather than as a single rigid partition. Drawing on four years of data from a large-scale photo-sharing platform, we show that certain hashtag combinations serve as stable anchors of cultural meaning, whereas others undergo substantial reorganization. By offering a robust methodological framework and new empirical insights, our work advances the quantitative study of cultural dynamics and, from a broader artificial-life perspective, contributes to understanding how large-scale interactions mediated by the web can give rise to emergent mesoscale structures in cultural ecosystems [7,29].

## 2. Methods

### 2.1. Data Collection and Processing

Our analysis uses hashtag data from RoomClip (RoomClip: https://roomclip.jp/ (accessed on 3 December 2025)), a Japanese photo-sharing social media platform focused on home interiors, furniture, household items, and DIY practices, where users share images of living spaces and annotate posts with hashtags.

The dataset, provided directly by RoomClip, consists of post records that include the timestamp, user ID, photo ID, and a list of hashtags attached to each post. In the present study, we focus on data from 2016 to 2019, a period of four years during which the platform had reached a more mature stage and posting activity remained relatively stable. This dataset comprises approximately 800,000 unique hashtags and 25 million hashtag occurrences across 3 million posts by 140,000 unique users. Reflecting the platform’s focus, the hashtags predominantly relate to living spaces and lifestyle choices, such as “#sofa,” “#fakegreen,” and “#DIY.” Consequently, the dataset captures a wide range of domain-specific cultural expressions and their temporal dynamics related to home trends and lifestyle preferences.

**Figure 1:**
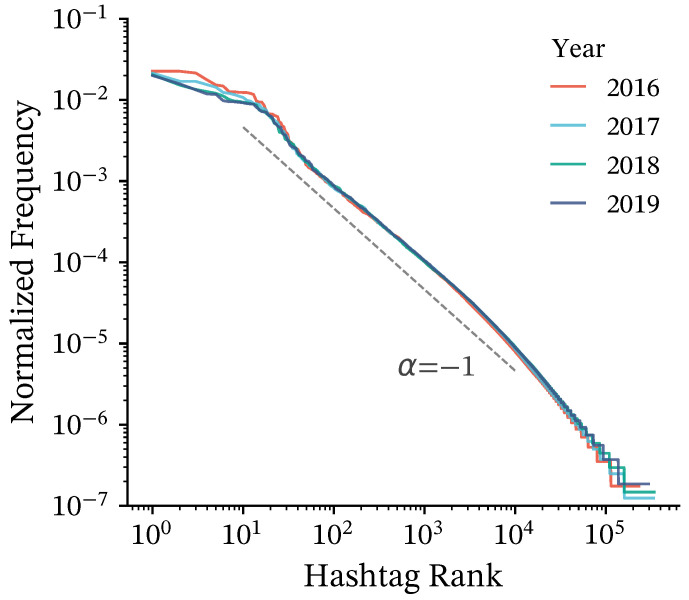
Rank–frequency distributions of hashtags for each year from 2016 to 2019. The distributions are highly skewed in all years, showing that a relatively small number of hashtags account for a large share of usage. A dashed line with slope α=−1 is shown as a guide to the eye, corresponding to a Zipf-like relation.

Figure 1 shows the yearly rank–frequency distributions of hashtags before filtering. In all four years, the distributions are strongly skewed, with a small number of highly frequent hashtags and a long tail of infrequent ones.

Given this highly skewed nature of hashtag usage, we implemented a systematic filtering process based on the number of unique users rather than raw occurrence counts. Specifically, we extracted hashtags used by at least 10 unique users in each year, ensuring that the selected hashtags consistently appeared throughout the four-year period (2016–2019). This filtering process yielded 9186 representative hashtags, constituting only 1.1% of the total unique hashtags recorded over the four years. Table 1 summarizes yearly dataset statistics and the annual coverage of this retained subset. Although the filtered hashtags represent only a small fraction of the yearly vocabulary, they account for 60.5–66.9% of all hashtag annotations in each year, indicating that hashtags adopted by a broad range of users dominate the platform’s overall discourse.

Because our focus is on changes across years in these widely used hashtags, we construct four independent yearly networks (2016–2019) rather than a single longitudinal network with finer temporal resolution. Given this coarse temporal resolution and the strong seasonal and event-driven shifts characteristic of social media platforms [30], we deliberately refrain from imposing an explicit temporal smoothing prior on community structure and instead compare ex post the independently inferred yearly modules [26].

### 2.2. Construction of Co-Usage Networks

We construct hashtag networks from the filtered dataset to capture semantic associations among tags. In this study, two hashtags are linked when they co-occur in the same post within a given year. The basic strength of each link is quantified by the number of unique users who have produced at least one post containing that hashtag pair, and we then derive edge weights from these co-usage counts based on unique users. This design combines co-occurrence at the post level with weighting at the user level, so that repeated posts by the same user do not artificially inflate the strength of associations.

**Figure 2:**
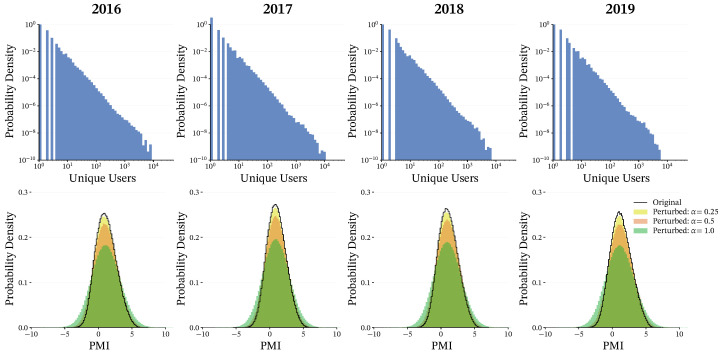
Probability density distributions of edge weights. (**Top**) Number of shared users per hashtag pair (log-log scale), showing a highly skewed distribution in which a few strong connections dominate. (**Bottom**) PMI distribution (black line) with overlaid perturbed versions with α=0.25 (yellow), 0.5 (orange), and 1.0 (green). Note that at α=0.25, the distribution essentially retains its original shape, indicating that the added noise does not disrupt the global statistical properties of the network.

For each year, we construct a separate network in which nodes represent the 9186 unique hashtags and edges represent post-level co-occurrence, with edge weights reflecting the overlap in the user base. Edge weights are defined by the pointwise mutual information (PMI) [31] calculated strictly within the filtered dataset. Let n(i) and n(j) denote the numbers of unique users who used hashtags *i* and *j*, respectively, and n(i,j) the number of users who have produced at least one post in which *i* and *j* co-occur. Furthermore, let *N* denote the total number of unique users associated with the set of 9186 hashtags in that year. We define the empirical probabilities

(1) $$p\left(i\right)=\frac{n(i)}{N},\;p\left(j\right)=\frac{n(j)}{N},\;p\left(i,j\right)=\frac{n(i,j)}{N},$$

and set the edge weight as

(2) $$w_{ij}=\log\frac{p(i,j)}{p(i)p(j)}$$

for all pairs with n(i,j)>0. Figure 2 illustrates why this normalization is important: the histogram of raw user co-occurrence counts (top) is heavy-tailed and exhibits large variance, indicating that a small number of extremely frequent pairs can dominate the weight scale if counts are used directly. In contrast, the black curve in the bottom panel shows the histogram of PMI weights, which forms a much more compact distribution, thereby mitigating the disproportionate influence of a small number of extreme edges. This PMI-based weighting highlights hashtag pairs that co-occur more often than expected by chance. By focusing on the user base, we can identify robust cultural modules that reflect shared interests and lifestyles among the platform’s community.

The resulting networks exhibit distinctive structural properties. Since we focus on widely adopted hashtags common across all periods, the networks comprise a fixed set of 9186 nodes (representing the intersection across all years). Among approximately 42 million possible hashtag combinations, we observe 2.0 to 2.6 million edges per year, indicating a densely connected structure. While PMI normalizes for usage frequency, the edge weights span a broad and continuous spectrum of association strengths. This high connectivity, coupled with the complex distribution of weights, poses unique challenges for module detection, necessitating robust analytical methods to separate genuinely stable association patterns from those that are sensitive to small perturbations in the edge weights.

### 2.3. Module Detection with a Perturbation Ensemble

To address the challenges posed by dense networks with heterogeneous edge weights, we employ a clustering method based on a perturbation ensemble. For each base network, we generate 1000 perturbed network realizations (ensemble members) by adding independent Gaussian noise with mean 0 and standard deviation $\sigma_{\mathrm{per}}$ to the PMI edge weights:

(3) $$w_{ij}^{\left(b\right)}=w_{ij}+\epsilon_{ij}^{\left(b\right)},\;\epsilon_{ij}^{\left(b\right)}∼N\left(0,\sigma_{\mathrm{per}}^{2}\right),$$

where *b* indexes the perturbed realizations and $\sigma_{\mathrm{per}}$ controls the strength of the perturbation. In practice, we set

(4) $$\sigma_{\mathrm{per}}=\alpha \sigma_{\mathrm{PMI}},$$

where $\sigma_{\mathrm{PMI}}$ is the empirical standard deviation of the PMI values across all existing edges in the base network and α is a dimensionless scaling factor. This choice ties the perturbation scale to the natural variability of association strengths in each yearly network: smaller values of α leave most partitions unchanged across perturbed realizations. We considered three perturbation strengths, α=0.25, 0.5, and 1.0, and present the corresponding results in the following section.

**Figure 3:**
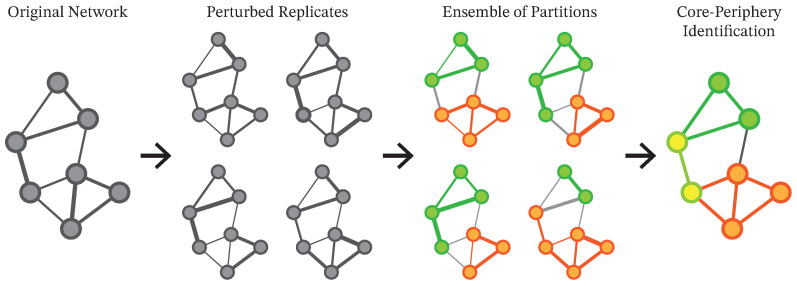
Schematic illustration of the perturbation ensemble clustering method. Gray nodes and edges represent the original network and its perturbed replicates. In the ensemble of partitions, green and orange indicate different modules. In the final core–periphery identification step, green and orange nodes belong to different core modules, whereas yellow nodes belong to the periphery.

We apply the Leiden algorithm [25], an improvement over the Louvain method [32], to each perturbed network realization as the base partitioner, using modularity maximization [33]. The Leiden method is particularly suitable for our analysis because it guarantees communities that are well connected internally and is computationally efficient for handling many large, dense networks. In this study, Leiden serves as a representative and well-established choice for large weighted graphs; our methodological interest lies in the perturbation ensemble layer used to assess robustness, rather than in benchmarking alternative partitioners. As illustrated in Figure 3, this process generates multiple clustering patterns for each perturbed realization. The resulting variation provides a basis for assessing the robustness of module structures. Rather than relying on a single hard partition for each yearly network, we obtain an ensemble of plausible partitions, summarized as node-level module assignment vectors and pairwise frequencies of being assigned to the same module, enabling us to quantify both module robustness and the uncertainty in individual hashtag assignments.

To complement the stability analysis based on the Hamming distance introduced below, we also summarize the ensemble partitions using two standard quantities: the modularity *Q* of each partition and the normalized mutual information (NMI) between partitions. Let $P^{\left(b\right)}$ denote the partition obtained from perturbed realization *b*. For the main setting α=0.25, we compute $Q(P^{\left(b\right)})$ for each ensemble member and $\mathrm{NMI}(P^{\left(b\right)},P^{\left(b^{\prime }\right)})$ for all unordered pairs $b<b^{\prime }$, and report their mean and standard deviation in Table 2; we also report the mean and standard deviation of the number of modules per partition. Here, *Q* summarizes the internal coherence of individual partitions, whereas pairwise NMI summarizes the similarity of module assignments across perturbed realizations, with values closer to 1 indicating stronger agreement between partitions.

### 2.4. Core-Periphery Structure Identification

**Figure 4:**
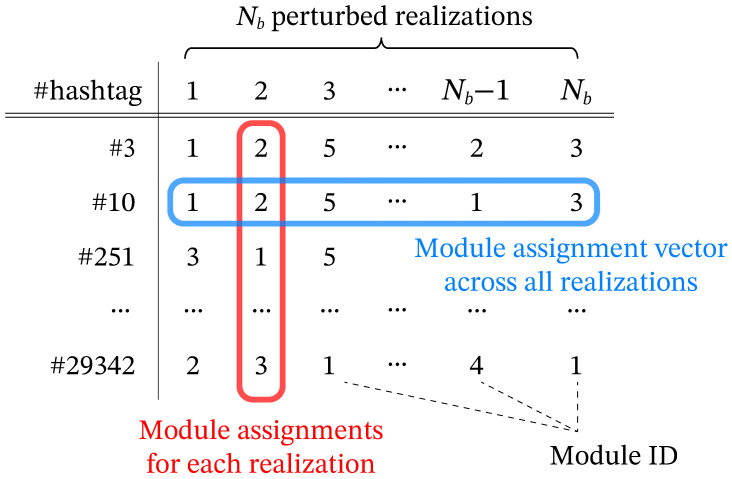
Module assignment vectors of each hashtag obtained from clustering results for all perturbed network realizations. Here, the symbol # denotes the prefix used in hashtag labels. The module IDs are assigned arbitrarily within each perturbed realization’s clustering, with no inherent correspondence across perturbed realizations.

The perturbation-ensemble clustering process generates a module assignment vector for each hashtag that represents its module assignments across all perturbed realizations (Figure 4). While module IDs are assigned arbitrarily within each perturbed realization, the similarity between module assignment vectors for hashtag pairs indicates an empirical tendency to be assigned to the same module under repeated weight perturbations.

We quantify this similarity using normalized Hamming distances between module assignment vectors, ranging from 0 (identical clustering patterns) to 1 (completely different patterns). From this perspective, a small normalized Hamming distance between two hashtags indicates that they are almost always assigned to the same module across perturbed realizations. In contrast, a large distance indicates hashtag pairs that are either weakly associated and sensitive to perturbations, or consistently belong to different contextual groupings.

**Figure 5:**
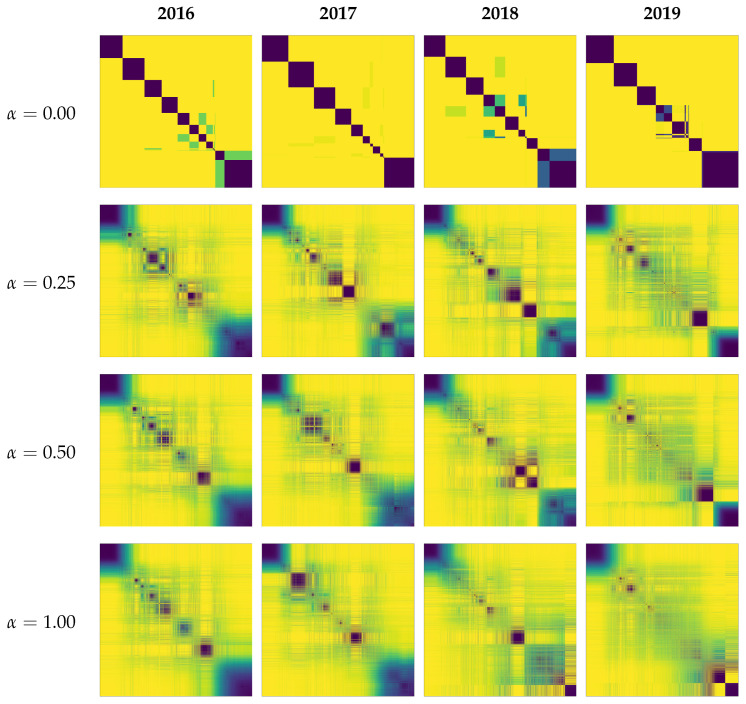
Heatmaps of the normalized Hamming distance matrix between all hashtag pairs. Darker colors correspond to lower normalized Hamming distances and brighter colors to higher normalized Hamming distances. Columns represent years (2016–2019), and rows represent perturbation strengths used in the clustering: α=0 (top), 0.25 (second), 0.5 (third), and 1.0 (bottom). While α=0 (almost equivalent to standard single-shot clustering) exhibits clear-cut block structures, even a slight perturbation (α=0.25) reveals fuzzy boundaries and ambiguous associations, thereby revealing the underlying fragility of the deterministic modular structure.

Figure 5 shows the matrix of the normalized Hamming distance between all possible pairs of hashtags for each year, with rows and columns sorted to group hashtag pairs with smaller distances together. In this heatmap representation, darker pixels indicate hashtag pairs with smaller distances (i.e., more consistent co-assignment across the ensemble), whereas lighter pixels indicate pairs with larger distances (i.e., less consistent co-assignment). Several darker blocks are observed in the matrix; their varying intensities reflect the modules’ sensitivity to noise. However, as the heatmap shows, even minimal perturbation reveals significant structural nuances. While the unperturbed case (α=0, top row) suggests rigid modularity (note that α=0 is also evaluated over 1000 Leiden runs; slight run-to-run differences may arise from algorithmic factors such as processing order or tie-breaking even without weight perturbations), the ensemble results with α=0.25 (second row) expose the inherent ambiguity of module boundaries. This contrast highlights that the seemingly “clear” partitions returned by a single run of clustering can be sensitive to minor fluctuations, motivating our ensemble approach to distinguish robust structures from fragile ones. Table 2 is consistent with this interpretation: under perturbation, modularity and the number of modules per partition remain stable, whereas pairwise NMI indicates only moderate agreement among partitions. Based on these distances, we define module structures as follows:

1. *Core*: A set of hashtags that form a fully connected clique, where every pair within the set is associated with a normalized Hamming distance $\leq \theta_{\mathrm{core}}$.
2. *Periphery*: Hashtags that maintain a normalized Hamming distance $\leq \theta_{\mathrm{core}}$ to at least one *Core* member, but are not part of the *Core* clique. We further classify these into:
  - *Private Periphery*: Hashtags associated with members of only a single *Core*.
  - *Bridge Periphery*: Hashtags associated with members of multiple *Cores* simultaneously.
3. *Floating*: Hashtags whose normalized Hamming distance to every *Core* member exceeds $\theta_{\mathrm{core}}$.

Here, we use the term “core” in a specific operational sense. While “core” may refer to different notions in network analysis (e.g., k-core decomposition), we adopt a stricter clique-based criterion in the similarity graph to isolate only highly robust cores under perturbations.

**Figure 6:**
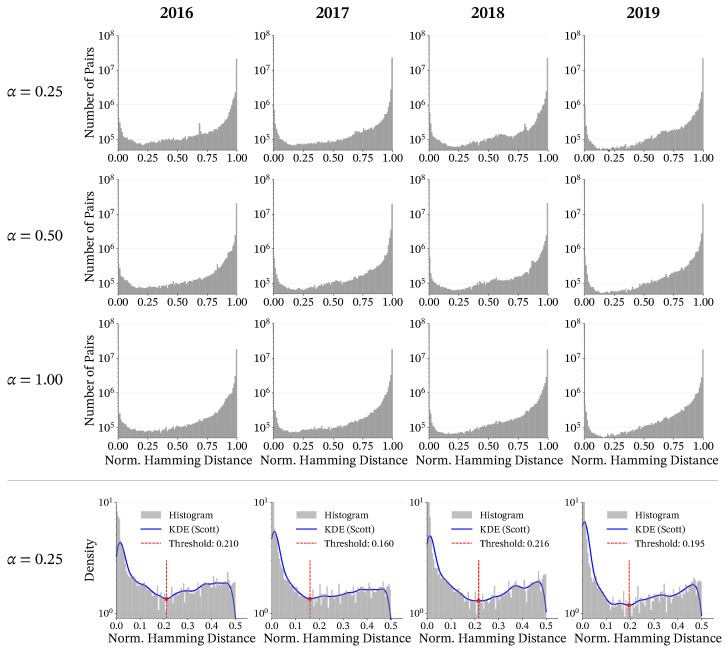
Histograms of normalized Hamming distances between hashtag pairs across years (columns) and perturbation strengths (rows). The top three rows display the raw histograms of normalized Hamming distances for α=0.25,0.5,and1.0, respectively. The consistent bimodal distribution across these values demonstrates the method’s robustness to parameter selection. The bottom row revisits α=0.25, now with a Gaussian kernel density estimation overlaid to identify the local minimum (red dashed line), which we define as the stability threshold $\theta_{\mathrm{core}}$.

In our implementation, we identify core members first and then assign peripheral members. To determine the core stability threshold $\theta_{\mathrm{core}}$, we smooth the histogram of normalized Hamming distances using Gaussian kernel density estimation (KDE) (Figure 6) and define $\theta_{\mathrm{core}}$ as the first local minimum of the KDE curve. The Hamming-distance distributions were qualitatively similar across the tested perturbation strengths, suggesting that the procedure is not overly sensitive to exact parameter choices. We therefore adopt α=0.25 for subsequent analyses: it provides enough perturbation to separate stable cores from unstable elements while limiting disruption to meaningful but weaker associations. Under α=0.25 (Figure 6, bottom row), this rule yields $\theta_{\mathrm{core}}=0.210$, 0.160, 0.216, and 0.195 for 2016–2019, respectively, giving a data-driven boundary that separates highly stable associations from more variable ones.

Then, we apply this same strict threshold $\theta_{\mathrm{core}}$ to identify peripheral members, rather than using a relaxed boundary (e.g., 0.5). Since our definition of the core imposes a strict topological constraint (i.e., complete connectivity in the similarity graph) and excludes nodes that are strongly connected but fail to form a clique, it is logical to define the periphery based on this topological distinction. Therefore, peripheral members are defined as nodes that possess the same high level of stability as core members but lack the complete connectivity required to be part of the core clique.

This classification provides a rigorous framework for identifying stable module structures. By enforcing the $\theta_{\mathrm{core}}$ threshold uniformly, we ensure that both core and peripheral members represent highly reliable cultural associations. *Core* members form the structural backbone, while peripheral members represent strong extensions of these clusters. *Bridge Periphery* members, in this strict context, act as strong inter-module hubs that bind distinct cultural contexts with high stability. *Floating* members are those that exhibit variable associations and are thus excluded from the stable structure.

## 3. Empirical Results

### 3.1. Visualization of Module Stability and Evolution

**Figure 7:**
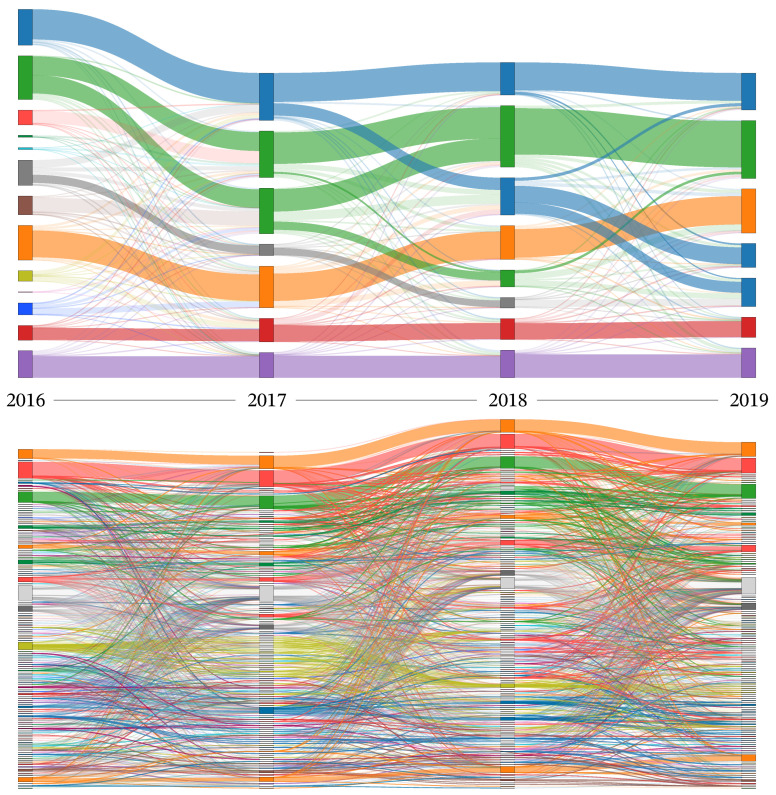
Comparison of temporal module evolution patterns between standard clustering and our perturbation ensemble approach. Each column represents a year (2016–2019). Nodes represent modules, and links connect modules in adjacent years that share hashtags; link width is proportional to the number of shared hashtags. In the top panel, nodes are modules obtained from the standard unperturbed Leiden clustering. In the bottom panel, nodes are *Core* modules identified by the perturbation ensemble approach, while *Bridge Periphery* and *Private Periphery* hashtags are aggregated into a single *Periphery* node (light gray), and the remaining hashtags into a single *Floating* node (dark gray). In both panels, module lineages are shown using a repeating 20-color palette, and links within the same lineage are drawn in the same color.

Before analyzing the detailed structural roles, we examine the global dynamics of module evolution to justify our ensemble approach. Figure 7 visualizes transitions between modules across yearly snapshots as a Sankey diagram, where each column corresponds to a year, each node to a module identified in that year, and each link to hashtags shared by modules in adjacent years. For clarity, we use two definitions of “module” corresponding to the two panels. In the standard baseline (top), modules are the Leiden communities obtained from the unperturbed yearly networks. In the ensemble-based visualization (bottom), modules correspond to the structural units identified as *Core* by the perturbation ensemble, while all *Bridge Periphery* and *Private Periphery* hashtags are aggregated into a single *Periphery* node and all remaining hashtags into a single *Floating* node.

The lineages shown in the figure are defined by relationships between consecutive years, based on the Jaccard overlap of module memberships, as described below. This comparison illustrates how a single run of clustering can mask structural fragility and ambiguity in cultural evolution [34].

To define module-level continuity across years under each definition, we compared every module in year *y* with all modules in the previous year y−1 using the Jaccard index of their member sets. For a module *A* in year *y* and a module *B* in year y−1, we computed

$$J\left(A,B\right)=\frac{\left|A\cap B\right|}{\left|A\cup B\right|}.$$

If J(A,B)≥1/3, we regarded module *B* as a parent module of *A*. This threshold is motivated by the definition of the Jaccard index: for two sets of equal size, J(A,B)=1/3 corresponds to half of their members overlapping. Note that a module can have multiple parents when it overlaps substantially with more than one module in the previous year.

Based on these relationships across consecutive years, we constructed and visualized module lineages, as shown in Figure 7, comparing temporal transitions derived from the standard unperturbed Leiden clustering (top) and our ensemble representation based on *Core modules* (bottom). In the standard approach (top), modules appear as distinct, continuous bands with sharp boundaries, suggesting a relatively simple and deterministic evolutionary path. However, this clarity is deceptive: the resulting partitions can be structurally fragile and may hinge on arbitrary algorithmic decisions in the presence of noise. In contrast, the ensemble-based visualization (bottom) emphasizes stable cores while making boundary ambiguity explicit through the *Periphery* and *Floating* aggregates. The resulting intricate web of transitions demonstrates that cultural structures do not merely persist or switch cleanly; *Core* modules may split or merge, and their membership can transiently diffuse into more ambiguous states. This comparison underscores the importance of capturing these “fuzzy” boundaries for a robust understanding of cultural dynamics.

### 3.2. Structural Roles and Community Scale

**Figure 8:**
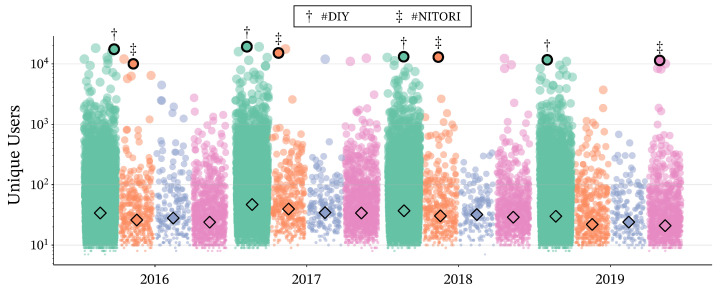
Distribution of unique user (UU) counts across structural roles (*Core*, *Bridge Periphery*, *Private Periphery*, and *Floating*) from 2016 to 2019. The jitter plot with median markers (diamonds) on a log scale demonstrates that *Core* members consistently capture the highest user attention.

To elucidate the relationship between structural centrality and community scale, we examined the distribution of unique user (UU) counts across the identified categories (*Core*, *Bridge Periphery*, *Private Periphery*, and *Floating*). Given the heavy-tailed nature of UU counts in social network data [35], Figure 8 shows these distributions on a logarithmic scale as a jitter plot with overlaid median markers.

As shown in Figure 8, the analysis reveals a distinct relationship between structural roles and popularity. Nodes classified as *Core* consistently exhibit high median UU counts, confirming that hashtags tightly integrated into specific cultural contexts drive significant engagement. Meanwhile, all non-*Core* categories (*Private Periphery*, *Bridge Periphery*, and *Floating*) show comparatively lower UU counts overall, indicating that hashtags outside the *Core* tend to attract less engagement. Nonetheless, each non-*Core* category contains a small number of exceptionally popular hashtags whose UU counts rival those of the most prominent *Core* members.

To illustrate this distinction, we highlight two contrasting examples in Figure 8: “#DIY” and “#NITORI.” “#DIY” consistently maintains its position within the *Core* across all four years with high user engagement. This stability suggests that DIY (Do-It-Yourself) constitutes a distinct and robust cultural genre that serves as a central pillar of the community. In contrast, “#NITORI” (a major furniture retailer) consistently resides in the *Bridge Periphery* (except in 2019) despite its comparable popularity. This suggests that such tags serve as generalist connectors: they link multiple distinct cultural modules (e.g., various interior styles) rather than being confined to a single specific niche. Thus, the *Bridge Periphery* position for popular tags implies a role of semantic diversity and cross-contextual connectivity, distinct from the thematic cohesion of the *Core*.

### 3.3. Temporal Dynamics of Structural Roles

**Figure 9:**
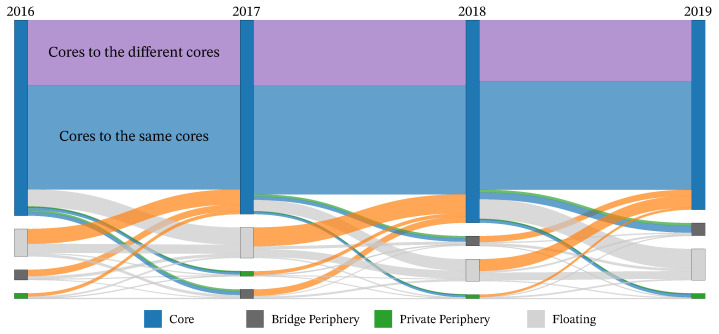
Temporal transitions of hashtags between structural roles. Flows represent the lineage of hashtags across consecutive years, colored by their source category. The thick horizontal flows within the *Core* category indicate strong self-preservation of cultural centers. In contrast, thick flows from one *Core* to another and intricate flows from the *Private Periphery*, *Bridge Periphery*, and *Floating* into the *Core* reveal the continuous metabolic turnover and reorganization of cultural communities.

We further investigated the stability and evolution of these structural roles over time. To do so, we aggregated hashtags by their structural category (*Core*, *Bridge Periphery*, *Private Periphery*, and *Floating*) for each year and visualized transitions between categories across consecutive years in Figure 9. In this diagram, flows represent hashtags moving between structural categories across adjacent years, and flow width is proportional to the number of hashtags following each transition.

The visualization highlights distinct dynamic properties across categories. The *Core* exhibits strong self-preservation, reflected in the thick “Same Core” flows, indicating that once a hashtag attains a central position, it tends to retain that status. At the same time, the diagram reveals the network’s metabolism: a persistent flux of upward mobility is observed, with hashtags from the *Bridge Periphery* or *Floating* moving into the *Core*, while former *Core* hashtags lose connectivity and drift toward the periphery. Moreover, distinguishing between “stable lineage” and “faction switching” (i.e., transitions into a structurally different *Core*) shows that although the *Core* category is stable in aggregate, the internal configuration of communities is continually reorganized—potentially through processes of merger and fission that dissolve existing *Core* modules and give rise to new *Core* structures over time.

## 4. Discussion

Taken together, the empirical results indicate that cultural organization on the platform is maintained through the coexistence of persistent cores, semantically connective peripheries, and ongoing turnover among more weakly attached elements. In this sense, the network exhibits neither a fully rigid modular structure nor complete fluidity. Rather, stable mesoscale organization is preserved under perturbation, while a non-negligible fraction of node assignments remains ambiguous and subject to reconfiguration. This suggests that cultural modules on the platform should be understood not as fixed partitions, but as evolving structures with robust centers and permeable boundaries.

From this perspective, the emergence, persistence, and reorganization of cultural modules should not be attributed solely to endogenous network dynamics; they may also reflect exogenous events and conditions. In this study, we interpret hashtag co-usage networks as observed cultural traces and ask which parts of that observed mesoscale organization remain robust under perturbation. *Bridge Periphery* hashtags associated with widely shared products, brands, or practices may connect otherwise distinct cultural contexts by reflecting common market actors or everyday material constraints. Likewise, changes in core and peripheral roles may reflect seasonal events, housing practices, or shifts in consumer attention. More broadly, recent work on collective-attention networks has suggested that economic output is associated with core and connector roles, while geographic proximity helps structure mesoscale communities [18]. Although the present dataset does not include external covariates such as regional economic indicators or spatial proximity measures, the framework developed here provides a basis for examining how such exogenous factors interact with endogenous cultural organization.

Future work will focus on the *Floating* states identified in this study. Rather than discarding these unstable elements, we aim to investigate them as the primordial soup of cultural innovation. Analyzing the semantic characteristics of hashtags that enter or leave these floating states could illuminate the mechanisms by which novel cultural elements are generated, tested, and eventually integrated into the stable core of the cultural lexicon. More generally, future work could relate floating states and module transitions to social, spatial, seasonal, and market variables, and also examine how the same perturbation framework behaves when combined with alternative base partitioners, since the present results are obtained within a Leiden-based partitioning framework.

## 5. Conclusions

This study presented a methodological framework for analyzing the temporal dynamics of cultural modules in hashtag co-occurrence networks, addressing the inherent challenges of noise and instability in social media data. By employing a perturbation ensemble clustering approach, we moved beyond the artificially rigid partitions of standard methods to reveal the fuzzy and complex reality of cultural evolution.

Applied to four years of hashtag data from a photo-sharing social media platform, the framework showed that apparently clear community boundaries are often structurally fragile under small perturbations and that these structural roles are continually reorganized across years. In particular, *Core* and *Periphery* represent distinct forms of stable cultural organization, whereas *Floating* captures more weakly attached and unstable elements. This suggests that cultural organization on the platform maintains its structural coherence through a dynamic balance between stabilizing specific contexts and expanding general connections. In this sense, the cultural meaning of hashtags is not fixed at the level of isolated tags, but is stabilized and reorganized through mesoscale patterns of association.

## Figures and Tables

**Table 1:** Summary statistics of the RoomClip dataset by year and coverage of the retained hashtag subset. Counts are computed separately for each year. The retained subset corresponds to hashtags used by at least 10 unique users in every year from 2016 to 2019.

	2016	2017	2018	2019
Yearly unique hashtags	231,701	335,193	340,092	296,395
Yearly active users	50,412	61,038	48,693	46,394
Posts	728,696	962,279	764,468	603,222
Total hashtag annotations	5,701,439	8,030,841	6,756,738	5,372,085
Annotations per post (mean ± SD)	7.8 ± 5.2	8.3 ± 5.6	8.8 ± 5.7	8.9 ± 6.1
Annotations from retained hashtags	3,816,108	5,230,254	4,183,575	3,250,650
Coverage of retained hashtags (%)	66.9	65.1	61.9	60.5

**Table 2:** Ensemble-level summaries for the main setting α=0.25. ‘Modules per partition’ denotes the number of detected modules in each perturbed partition, reported as mean ± standard deviation across the 1000 ensemble members. Modularity *Q* and pairwise NMI are likewise reported as mean ± standard deviation.

Year	Modules per Partition	Modularity *Q*	Pairwise NMI
2016	10.5±2.6	0.213±0.003	0.599±0.054
2017	11.4±2.9	0.203±0.003	0.634±0.060
2018	11.6±2.5	0.202±0.002	0.622±0.062
2019	11.7±3.5	0.204±0.002	0.602±0.055

## Data Availability

The raw data used in this study cannot be made publicly available due to contractual restrictions. Processed data supporting the reported results are available from the corresponding author upon reasonable request, subject to the applicable contractual limitations.
